# Proteomic and Machine Learning Analysis Predicts Treatment Response Signatures in Myasthenia Gravis

**DOI:** 10.21203/rs.3.rs-7915342/v1

**Published:** 2025-11-26

**Authors:** Karli Faith Gilbert, Amrita K. Cheema, Henry Kaminski, Linda Kusner

**Affiliations:** George Washington University; Georgetown University Medical Center; George Washington University; George Washington University

**Keywords:** myasthenia gravis, machine learning, treatment response, proteomics, mass spectrometry, prednisone, thymectomy

## Abstract

**Background:**

Myasthenia gravis (MG) is a prototypical antibody-mediated autoimmune disease with variable treatment responses with a need for biomarkers to guide therapeutic decision making. Proteomic profiling, coupled with machine learning, offers a powerful approach to identify biomarkers that may predict treatment response.

**Methods:**

We analyzed sera collected at entry (baseline) from participants in a phase 3 trial randomized trial comparing thymectomy plus prednisone versus prednisone alone, along with matched controls using liquid chromatography–mass spectrometry. We derived disease-specific proteomic signatures and evaluated associations between baseline proteins and 6-month clinical outcomes using multiple machine-learning approaches with internal validation.

**Results:**

Baseline serum proteomes distinguished MG from controls, with pathway enrichment implicating complement activation, immunoglobulin production, and T-cell receptor signaling. Distinct protein panels predicted 6-month clinical improvement within each treatment arm. In the thymectomy-plus-prednisone group, models captured non-linear relationships of predictive proteins in contrast with the predominant additive patterns observed in the prednisone-alone group. Predictive proteins were enriched for T-cell signaling and leukocyte trafficking functions, providing insight into treatment-specific biology.

**Conclusions:**

Baseline serum proteomics captures core disease characteristics of MG and predicts short-term clinical response in a treatment-specific manner. If validated in independent cohorts, these findings could enable biomarker-guided selection of thymectomy, refine risk stratification, and furnish mechanistic readouts for future MG trials and clinical care.

## Background

Therapeutic decision-making for patients with autoimmune diseases is often hindered by the central challenge of the unpredictability of treatment response. Whether due to intrinsic disease heterogeneity or variable sensitivity to immunomodulatory therapies, patients with the same diagnosis can experience markedly different outcomes. The complex interplay of many factors, including sex, age, genetic background, and environmental experiences, collectively influence disease expression and therapeutic efficacy. Myasthenia gravis (MG), a prototypical antibody-mediated disorder, exemplifies this clinical variability and offers a compelling model in which to investigate the biological underpinnings of treatment response and identify biomarkers to predict therapeutic response (1, 2).

Despite an array of groundbreaking research in the field of MG, few studies have combined rigorous, long-term clinical outcome monitoring with systematic collection of linked biological specimens. A notable exception is the NIH-supported, Thymectomy Trial in Non-Thymomatous Myasthenia Gravis Patients Receiving Prednisone Therapy (MGTX) trial, which incorporated comprehensive, validated clinical assessments linked with prospectively collected biospecimens. The MGTX trial, with a primary endpoint at 3 years, established the efficacy of thymectomy plus prednisone (ETTX) compared with prednisone alone (PA) in patients aged 18 to 65 years with acetylcholine receptor (AChR) antibodypositive MG (3). Subjects in the thymectomy arm had a lower total Quantitative MG (QMG) score and required lower overall corticosteroid dosage at end of study, although no significant differences were detected at 6 months. Prednisone is a well-established therapy for MG (4, 5), while the application of thymectomy dates back nearly 100 years, but was only unequivocally identified as effective by the MGTX trial. However, the mechanisms underlying the benefit of thymectomy remain poorly understood. Considerable variability in treatment response was observed across both study arms.

Broad-based -omics profiling approaches provide a powerful method to move beyond clinical observation toward molecular signatures that predict therapeutic outcomes. Optimization of treatment regimens in patients with MG can be supported by the identification of biomarkers predictive of treatment outcomes (6, 7), a task increasingly enabled by machine learning approaches applied to clinical features and multi-omics data. Studies applying machine learning to investigate biospecimens from MG patients are limited and have used publicly available microarray expression profiles (8–10), while others have generated primary proteomic (11), metabolomic (12), and transcriptomic data (13) from MG cohorts.

Here, we applied liquid chromatography–mass spectrometry (LC-MS) to sera collected at study entry (baseline) from 86 subjects of MGTX trial, of whom 41 were assigned to PA and 45 to ETTX. We first assessed for differences between MG subjects and controls and then aimed to identify serum proteins at baseline predictive of 6-month QMG improvement in treatment groups. Although subjects who had undergone thymectomy demonstrated a greater average reduction in QMG scores compared to prednisone-only subjects, about 30 percent of subjects did not achieve a well-established criterion of a clinically meaningful reduction of 3 points on the QMG score in the first 6 months of the study. We chose this early time point because of the greater uniformity of treatment early in the study based on uniform prednisone treatment protocol, an absence of rescue therapies, and expectation of thymectomy providing an early effect on the autoimmune pathology of MG. We made no prior assumptions regarding the data set and applied several machine learning (ML) models for predictors of treatment response.

## Methods

### Study Population

Serum samples were obtained from participants enrolled in the MGTX clinical trial (NS42685). Specimens had been stored at −80°C at NCI-Fredrick and then transferred to the biospecimen bank at George Washington University. Samples were collected at the baseline time point for each participant and analyzed using LC-MS. Clinical outcome measurements were collected at baseline and six months using the QMG assessment. All individuals included in this analysis had confirmed diagnosis of MG with detectable acetylcholine-receptor autoantibodies and no evidence of thymoma (3). Control serum samples were collected at George Washington University with the exclusion criteria of autoimmune disease and vaccination in the past month. All participants were older than 18 years and provided written informed consent. Patient and control characteristics are summarized in Supplemental Table 1.

The NINDS funded the trial and assembled an independent Data Safety Monitoring Board. Sites received local institutional review board (IRB)/ethics committee approvals, and each patient provided written informed consent before study entry including provision of serum samples. All specimens were deidentified. The George Washington University IRB provided additional review and approved use of specimens.

#### Mass Spectrometry Protein Analysis

Shotgun proteomics of MG patient sera was performed as previously described (14, 15). Individual samples (20 μL) and a pooled quality control (5 μL of each sample) were prepared using ENRICH-iST Kit (PreOmics^®^) before injection into the mass spectrometer nanoElute^®^ 2 instrument (Bruker Scientific). Peptide fractions from the pooled quality control (9 technical replicates) were obtained using the Pierce^™^ High pH Reversed-Phase Peptide Fractionation Kit. A data dependent acquisition (DDA) spectral library from the peptide fractions was generated in Fragpipe and DIA_SpectLib_quant workflow. Individual samples were quantified with DIA-NN (data independent acquisition – neural network) with a maximum false discovery rate of 1%.

#### Data Cleaning and Imputation

The DIA quantification values were first filtered in Fragpipe (16) to remove contaminant proteins, reverse sequences, and proteins identified by a single peptide. The data were then converted to a log2 scale and separated by group (MG vs Control). Missing values were imputed using a Perseus-type Missing Not at Random (MNAR) approach, where values were randomly drawn from a left-shifted Gaussian distribution. This distribution was centered 1.8 standard deviations below the mean of the observed values within each group, with a standard deviation width of 0.3, to simulate low-abundance proteins below the detection limit.

#### Analysis of Myasthenia Gravis versus Control Subjects

The R package *limma* was used to determine the differentially expressed proteins (DEPs) between MG and healthy control (HC) samples (17, 18). DEPs were defined as proteins that surpassed a significance cutoff of a |log_2_ fold change| >1 and an adjusted p-value less than 0.05 (Benjamini-Hochberg method). Pathway enrichment analyses were performed using over representation analysis (ORA) and Gene Set Enrichment Analysis (GSEA) with the R packages *clusterProfiler* and *enrichPlot* (19, 20).

#### Feature Selection

We applied the Boruta feature selection algorithm to identify all relevant variables for predictive detection of treatment predictive proteins. The approach enables optimization of a low sample/feature ratio, to reduce the number of proteins, and thus noise, in our predictive analyses. This allowed for enhanced performance in downstream assessments and reduced the likelihood of a classification algorithm from overfitting a model (21).

### Boruta

The Boruta algorithm (22) was employed in R using the *Boruta* package with default settings, *maxRuns* = 300, and results recorded from 100 iterations, to obtain a subset of proteins predictive of clinical improvement in thymectomy plus prednisone (ETTX) and prednisone alone (PA) subjects separately. In each iteration, Boruta creates shadow features that are added to the dataset, runs a single random forest model on the dataset and compares the importance scores of the real variables to the shadow features. Features whose importance scores were significantly higher than the highest importance score observed among the random noise variables were retained as relevant predictors. The initial subsets of proteins were then subjected to a second set of 100 iterations of Boruta feature selection to obtain a set of proteins for PA and ETTX patients for use in nested cross validation of eight ML models.

#### Nested Cross Validation

Nested cross-validation (nCV) was employed to separate model training from testing, ensuring unbiased performance estimates and reducing the risk of overfitting in clinical outcome predictions. We separately applied a 5-fold nCV to the selected features for ETTX and PA subjects. The inner loop performed hyperparameter tuning via grid search (Supplemental Table 2) using the *caret* R package with its default 10-fold CV to determine the most accurate training parameters for the outer loop. The data were split across the outer folds such that each patient was tested once. Classification predictions and performance metrics were pooled or averaged across outer folds to generate unbiased estimate.

#### nCV Models

To develop robust and generalizable predictive models, we employed a diverse panel of supervised ML algorithms within the nCV framework: probabilistic classifiers (Naïve Bayes, Logistic Regression), regularized linear models (LASSO, Elastic Net), tree-based approaches (Classification Tree, Random Forest), and ensemble boosting methods (AdaBoost, XGBoost). Each model captured different aspects of data structure, which allowed us to comprehensively evaluate predictive features across varying assumptions and model complexities. This broad approach was chosen to minimize bias from any single modeling strategy and to better characterize proteomic expression patterns relevant to predicting clinical improvement.

### Probabilistic Classifiers

We implemented Naïve Bayes and logistic regression as representative probabilistic classifiers. Naïve Bayes, which assumes independence among features, was applied using the *naiveBayes* function from the *e1071* R package (23). Logistic regression, which models the log-odds of the binary outcome as a linear combination of predictors, was performed using the base R *glm* function with a binomial family. Both models are deterministic and reproducible, in contrast to stochastic tree-based and ensemble methods.

### Regularized Linear Models

LASSO (Least Absolute Shrinkage and Selection Operator) is a linear regression technique that uses L1 regularization (*α* = 1) to shrink the coefficients of less important features to zero. Similarly, elastic net is a regularized regression that balances L1 and L2 regularization (0 < *α* < 1). We performed LASSO and Elastic Net using the *glmnet* R package (24, 25). Using the built-in cross-validation, proteins with nonzero coefficients were recorded from two regularization levels: *λ*min, the value of *λ* that minimized the cross-validation error, and *λ*1se, the largest λ within one standard error of *λ*min. These correspond to models that favor predictive accuracy and model simplicity, respectively.

### Decision Trees

Deterministic decision trees were made using the classification and regression tree algorithms (CART) from the recursive partitioning and regression tree R package *rpart* (26). For each split at a node, the relative impurity reduction was calculated as the impurity at the parent node minus the total impurity of the left and right child. A named numeric vector for each tree provided the protein names of those considered important by the algorithm.

### Random Forests

The random forests algorithm was performed using the R package *randomForest* (27), which ranks features based on how much they contribute to decreasing the variance in the model, also referred to as the increase in node purity. The importance scores calculated for each protein are a normalized calculation of the contribution of each protein to the reduction of impurity in the model.

### Ensemble Boosting

Adaptive boosting (AdaBoost) and extreme gradient boosting (XGBoost) were used as ensemble learning methods, which work to improve predictive performance by sequentially training models, each focused on correcting the errors of the previous. AdaBoost was implemented using the *ada* function from the *JOUSBoost* R package (28), while XGBoost was applied using the *xgboost* package (29). Both methods iteratively reweigh training observations to emphasize harder-to-classify cases. A key distinction is that AdaBoost used simple decision stumps as base learners, whereas XGBoost built full decision trees, allowing it to capture more complex patterns.

A complete list of R packages and versions used can be found in Supplementary Table 3. Statistical analyses were performed in R v4.4.1 and GraphPad Prism 10.3.0. The repository with all R source code used to run nCV is available on GitHub at https://github.com/drkgil/NestCV.

### Pathway and Tissue Source Analysis

We performed GeneAgent analysis (30) to assess biological pathways identified for proteins found to be predictive for outcomes for PA and ETTX groups.

## Results

### Proteomic profiling reveals distinctions between MG patients and controls.

**Figure 1:**
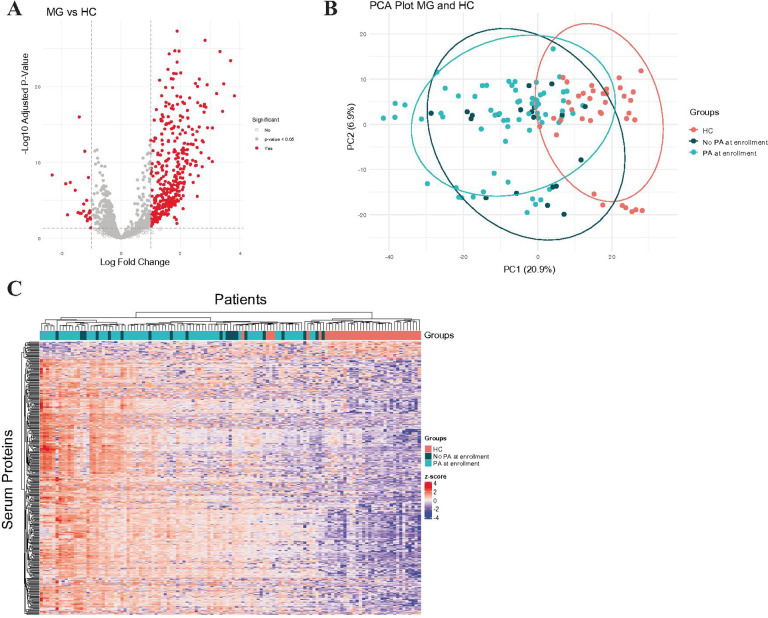
Patients with MG have distinct serum proteomic profiles compared to non-autoimmune controls. A. Volcano plot of 1,210 detected serum proteins in MG patients relative to controls. Significant proteins (red) are those that passed cutoffs (dotted lines) of > 1 or < −1 log2 fold change and less than the −log10 adjusted p-value of 0.05. B. PCA plot of all patients using all 1,210 proteins. Ellipses represent corresponding 95% confidence intervals for each healthy controls (pink), MG patients on prednisone (blue), and MG patients not on prednisone (dark blue) at the time of study enrollment. C. Unsupervised clustered heatmap of 398 DEPs (Euclidean distance and complete-linkage clustering). Heatmap was created using *pheatmap*.

Eighty-six sera at study entrance to the MGTX clinical trial and 37 healthy controls were used (demographic details in Supplemental Table 1). While efforts were made to match the groups, the average age of subjects differed from controls (Control: 43.46 years; MG: 35.91 years; Mann-Whitney U = 1090, p = 0.0053); there was no difference in gender distribution. We identified proteomic signatures differentiating MG patients from controls. Liquid chromatography mass spectrometry analysis identified 1,213 proteins, from which a dataset of 1,210 were used to identify 398 differentially expressed proteins (DEPs). Among these, 23 proteins were significantly downregulated in MG compared to controls, while 375 proteins were significantly upregulated, highlighting a substantial alteration in the proteomic landscape associated with MG (Fig. 1A).

Principal component analysis (PCA) of the full proteomic dataset further revealed a clear separation between MG and control samples, emphasizing the robustness of these differences (Fig. 1B). Among the MG patients, 77% were on prednisone at the time of study enrollment at an average dose of 30.7 mg ± 14.1 (Supplemental Table 1). We found that the use of prednisone at the time of enrollment was not a driver of the PCA separation from controls. Hierarchical clustering of the 398 DEPs further supported this distinction, with heatmap visualization clearly separating MG patients from controls, without a pattern according to prednisone use (Fig. 1C).

**Figure 2:**
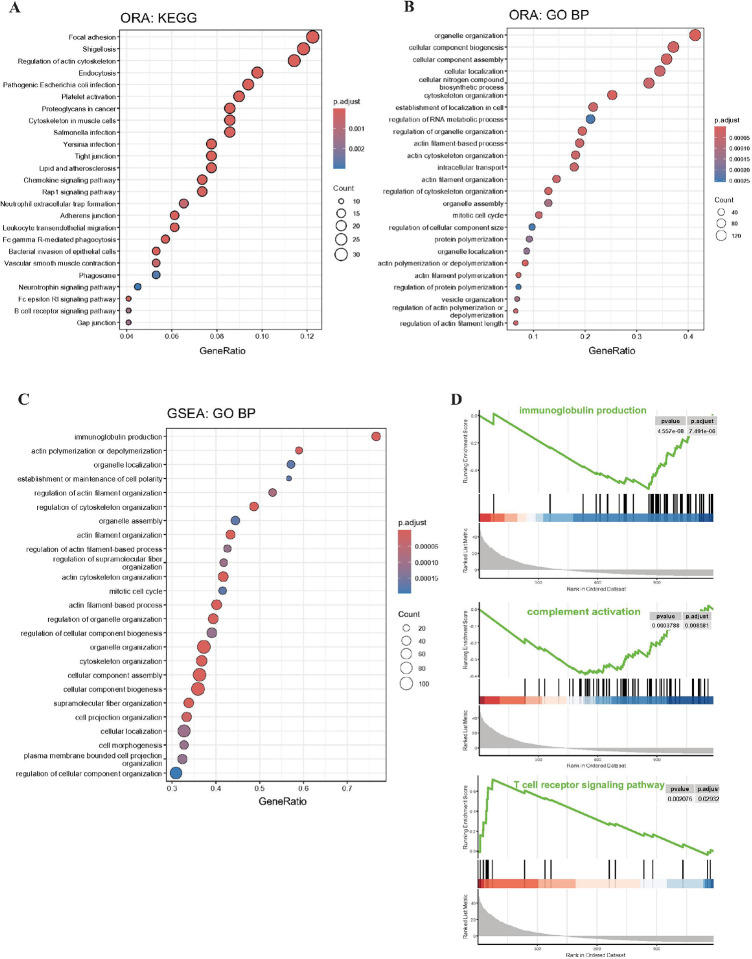
Pathway enrichment analyses indicate significant changes in immune relevant pathways. The order of the dots is presented according to the pathway gene ratio score, the color represents the BenjaminiHochberg (BH) adjusted p-value, and the size represents the number of genes from the dataset that matched the pathway annotation. A. Dot plot of the 25 most significant Over Representation Analysis (ORA) of the Gene Ontology Biological Process (GO: BP) pathways. B. Dot plot of the 25 most significant ORA of Kyoto Encyclopedia of Genes and Genomes (KEGG) pathways. C. GSEA dot plot of the top 25 most significantly enriched GO-BP pathways in MG patients. D. GSEA score plots immunoglobulin production (GO:0002377, p = 7.9e-5), complement activation (GO:0006956, p = 0.009), and T cell receptor signaling pathway (GO:0050852, p = 0.03).

To explore the biological processes associated with the 398 DEPs identified in MG, we performed ORA using the Gene Ontology Biological Processes (GO-BP) pathway database. The analysis revealed numerous significantly enriched pathways, with the top 25 pathways included those related to actin filament organization and organelle organization (Fig. 2A). ORA using the KEGG database mirrored the GO-BP results, with enrichment of terms related to actin organization and cytoskeletal dynamics (Fig. 2B). Several of these top KEGG pathways corresponded with those typically enriched in MG patients compared to healthy controls: “leukocyte transendothelial migration”, “B cell receptor signaling pathway”, and Fc receptor pathways.

To test for coordinated expression patterns that could be informative of changes between MG subjects and controls that may have escaped the threshold of significance set for DEPs, we also performed GSEA utilizing the GO-BP database. Proteins were ranked according to the B-statistic obtained from the *limma* analysis of Fig. 1. While many of the top pathways overlapped with those identified by ORA(Fig. 2C), a few significant pathways emerged. Notably, enrichment scores for immunoglobulin production (p = 7.5e-6) and complement activation (p = 0.0086) were significantly downregulated in MG patients compared to controls, while the T cell receptor signaling pathway was significantly upregulated (p = 0.03) (Fig. 2D).

### Distinct proteomic signatures emerge when prednisone and thymectomy treatment groups are analyzed separately

We next sought to determine which proteins could be a predictive marker of clinical improvement at 6 months based on a greater than three point improvement of the QMG score, which has been established as a clinically meaningful difference (31). The patient characteristics at baseline did not differ between the treatment groups (Supplemental Table 4).

**Figure 3:**
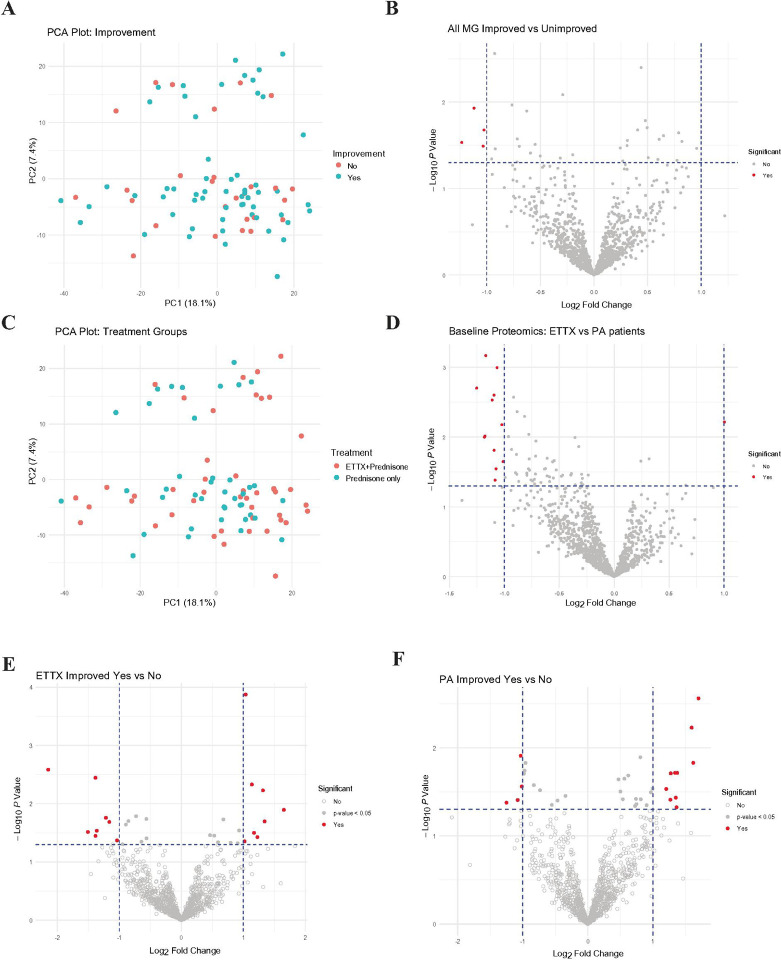
Baseline ETTX and PA differences between those who improved versus those who did not. Significance threshold of p-value < 0.5 and |log-fold change| > 1. A. PCA plot of all MG patients using all 1,210 proteins. Colors indicate if the patient improved in their QMG score at 6 months or not. B. Volcano plot of all proteins in patients that improve at 6 months in relation to those who did not improve. C. PCA plot of all MG patients, using all proteins, colored according to treatment group assignment, prednisone alone (blue) or thymectomy plus prednisone (pink). D. Volcano plot of all proteins in patients assigned to thymectomy plus prednisone versus patients receiving prednisone alone. E. Volcano plot of all proteins from ETTX patients who improved compared to ETTX patients that did not improve. F. Volcano plot of all proteins from PA patients who improved compared to PA patients that did not improve.

PCA of MG subject proteomics did not reveal a distinction between patients that improved compared to those that did not improve (Fig. 3A); however, four proteins were significantly reduced in patients that showed improvement (Fig. 3B). Similarly, the same PCA labeled by treatment group indicated a lack of clustering (Fig. 3C), despite DEPs present between ETTX and PA treated patients (Fig. 3D). This finding is not surprising given that the clinical trial did not show statistically significant differences between groups until after 6 months.

Next, we analyzed baseline serum protein profiles separately for each treatment group. Within the ETTX and PA treatment groups, comparisons between patients who improved and those who did not revealed 16 DEPs in ETTX patients (Fig. 3E) and 14 DEPs in PA patients (Fig. 3F). Given the greater number of DEPs identified within individual treatment groups compared to the combined MG subjects, we maintained this separation in the data analysis.

### Feature selection highlights proteins predictive of improvement and distinct between treatment groups

Considering the positive response of patients to thymectomy, we hypothesized that proteins predictive of clinical improvement would differ between treatment groups. The extent and nature of these differences was initially unclear as potential group-level differences were masked by the abundance of non-significant proteins, as seen in the PCA plots (Fig. 3A, C).

**Figure 4:**
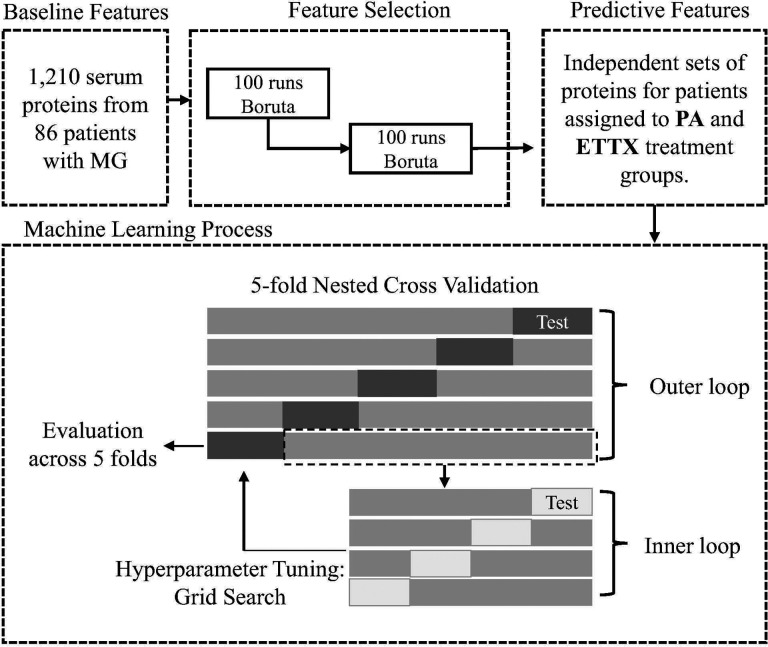
Feature selection and nested cross validation workflow.

To address this, we independently applied Boruta feature selection within each treatment group (Fig. 4), identifying preliminary sets of 45 proteins in ETTX patients and 38 proteins in PA patients. A second round of feature selection was then applied to these subsets to refine the list to proteins strongly associated with improvement. This two-step selection process reduced dimensional noise, yielding 17 and 7 key proteins for the ETTX and PA groups, respectively (Table 1). At each step of the feature selection, we verified that the sets of features were specific to its respective treatment group by performing PCA with mismatching the patient group and predictive proteins (Supplemental Fig. 2). These group-specific protein sets were used in all subsequent analyses to test their performance across eight different machine learning algorithms in a nested cross validation design.

### Random Forest identifies seven proteins highly predictive of response to prednisone treatment

We applied a five-fold nCV framework utilizing eight machine learning models, which decreases the potential for bias in the selected panels, on the relative abundances of 7 proteins, CCL16, CILP2, GALNT1, IGHV3–43, RPS3, RPS3A, and VAPB, expressed in PA patients at baseline. nCV is particularly well-suited for smaller datasets, as it helps prevent overfitting by ensuring each sample is independently tested from the data used to train the model (Fig. 4).

**Figure 5:**
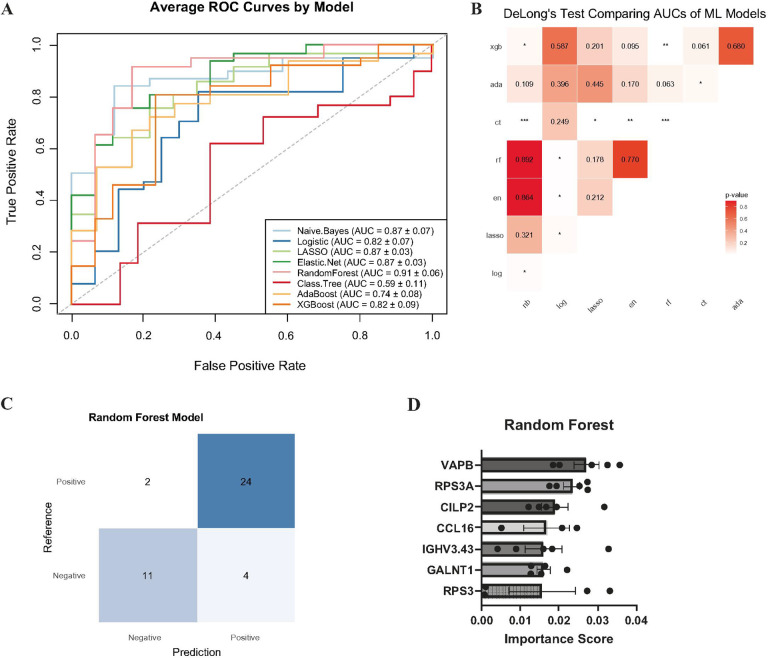
Random Forest model of 7 proteins in PA-treated patients was the best predictor of improvement. A. Comparison of ROC curves between 8 different machine learning models, averaged across five outer-folds. Model’s AUC mean and SEM are reported in the legend. The dotted gray diagonal line represents hypothetical performance of a model that classifies at random. B. Heatmap of p-values from DeLong’s test, which compares if the AUCs between two ROC curves generated from different ML model predictions are statistically different from one another. C. Confusion matrix of the random forest algorithm, using summed classification results across the outer five-folds from the nCV. D. Feature importance scores histogram of the 7 predictive proteins (mean ± SEM). Each dot represents the importance score from one individual outer fold. CCL16 was not deemed important in 2 of the outer folds and RPS3 in 1 of the outer folds, which is why they each have 3 and 4 dots on the graph, respectively.

Each model performed relatively well at classifying PA patients as improved or not, except for the classification tree algorithm. Several models showed similar performance, with receiver operating characteristic (ROC) curves and average area under the ROC curve (AUC) closely overlapping (Fig. 5A). However, differences between models were more evident when considering the Matthews correlation coefficient (MCC) (Table 2), a robust metric that accounts for all confusion matrix categories. MCC scores range from − 1 to 1, indicative of total disagreement and total agreement between predicted and actual classes, respectively (Chicco and Jurman, 2020). Together, the AUC and MCC scores highlight the random forest algorithm as the best-performing classifier for PA patients in the nested cross-validation.

The random forest algorithm produced the highest AUC 0.91 ± 0.06, which was significantly better than the logistic regression (Z = −2.48, CI −0.317 to −0.037, p = 0.013), extreme gradient boosting (Z = 2.89, CI = 0.043 to 0.23, p = 0.0039), and trended towards being better than adaptive boosting (Z = 1.86, CI = −0.006 to 0.216, p = 0.063) (Fig. 5B). Across the outer folds of the nCV, the RF model correctly classified 35/41 patients (Fig. 5C). The mean decrease in accuracy was used to determine which of the 7 proteins were most important to the RF model in accurately predicting patient improvement outcome. The protein *VAPB* had the highest average importance score, followed by *RPS3A*, and *CILP2* (Fig. 5D).

### Seventeen proteins predict treatment response to thymectomy

**Figure 6:**
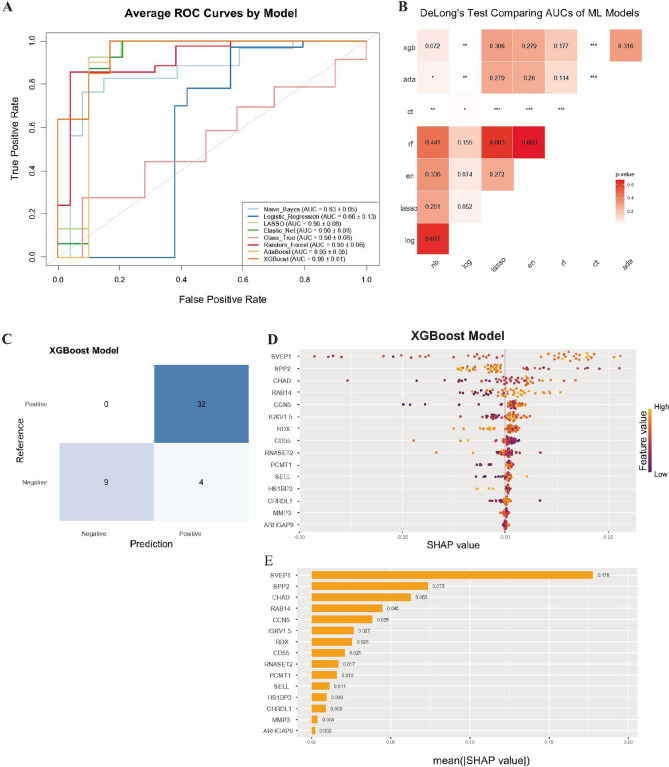
Extreme Gradient Boosting model of 17 proteins was best predicting improvement in ETTX-treated patients. A. Comparison of ROC curves between 8 different machine learning models, averaged across five outer-folds. Model’s AUC mean and SEM are reported in the legend. The dotted gray diagonal line represents hypothetical performance of a model that classifies at random. B. Heatmap of p-values from DeLong’s test, which compares if the AUCs between two ROC curves are statistically different from one another. C. Confusion matrix of the XGBoost algorithm, using summed results across the outer five-folds from the nCV. D. SHAP bee swarm plot for the XGBoost model, providing a visual explanation of how the most influential proteins contributed to the model’s predictions. Each dot is representative of one patient and its color corresponds to the value of that protein. E. SHAP importance plot of the features ranked from most to least important. The mean absolute value of the SHAP value is reported.

Similarly, we applied five-fold nCV to the 17 proteins predictive of improvement outcome in ETTX patients: ARHGAP9, CCN5, CD55, CHAD, CHRDL1, FBLN5, HS1BP3, HSP90AA1, IGKV1–5, MMP3, PCMT1, RAB14, RDX, RNASET2, SELL, SPP2, and SVEP1. The ROC curve analysis indicated that the adaboost and XGBoost models performed best, followed closely by LASSO, Elastic Net, Random Forest, and Naïve Bayes (Fig. 6A, Table 3). The XGBoost model achieved the highest AUC (0.99 ± 0.01), performing significantly better than Logistic Regression (Z = −2.67, CI = −0.37 to −0.06, p = 0.008) and Classification Tree algorithms (Z = −5.0, CI = −0.68 to −0.30, p = 5.7e-7), and trended towards significance over Naïve Bayes (Z = −1.80, CI = −0.34 to 0.15, p = 0.072) and Random Forest algorithms (Z = −1.35, CI = −0.23 to 0.04, p = 0.18) (Fig. 6B). Across all five outer folds of the nCV, XGBoost correctly classified 41/45 ETTX patients (Fig. 6C).

SHAP (SHapley Additive exPlanations) values were calculated to explain the XGBoost model. These values correspond to how much the protein contributes to the prediction in each individual; the greater the distribution of a protein’s SHAP values, the greater its influence on the prediction of the model. Since the model performs a binary classification for each patient, positive SHAP values correspond to an increased probability of being classified as ‘improved’; conversely, negative values correspond to an increased probability of being classified ‘not improved’ (Fig. 6D). In ETTX patients, the XGBoost model identified SVEP1, SPP2, CHAD, and RAB14 as the top important proteins for predicting clinical improvement (Fig. 6E).

### GeneAgent Analysis

We applied GeneAgent (30) analysis to the ETTX and PA proteins identified as predictors of clinical outcomes (Supplementary Files 6 & 7). The summary report for ETTX indicated that the extracellular matrix remodeling was the primary pathway involved in treatment response, with key indicators from cell adhesion, migration and immune response. In contrast, analysis of the seven proteins predictive of PA response revealed some association with ribosomal pathways, but no distinct summary was identified.

## Discussion

To better understand the immunobiological underpinnings of treatment response in MG, we performed serum proteomic profiling of participants in the MGTX clinical trial and healthy controls. We identified distinct serum proteomic signatures that differentiated individuals with MG from controls, with biological pathways involving complement activation, immunoglobulin production, and T cell signaling driving these differences. Moreover, we discovered a subset of proteins that predicted clinical improvement six months later, differing between subjects treated with prednisone alone and those receiving thymectomy plus prednisone. These findings provide unique insights into the biological effects of thymectomy. Notably, application of multiple machine learning approaches revealed non-linear relationships in the identification of outcome-predictive proteins in the ETTX plus prednisone group, while independent linear (additive) patterns predominated among those receiving prednisone alone. The proteins associated with treatment response were primarily involved in T cell signaling and cell trafficking.

The limitations of our work lie in those inherent to a rare disease research of small sample size, and despite our machine learning nested cross validation approach, future validation studies will be required, including those that assess change in the proteome over time. Further, the benefit of a rigorous clinical trial data set compromises the potential for immediate applicability to a real-world population with a greater diversity of patients with multiple co-morbidities. This includes patients outside the age of study entrance criteria and AChR antibody status.

### Myasthenia gravis driven differences from controls

Our initial hypothesis was that prednisone treatment would significantly alter the serum proteome and potentially confound disease-specific differences; however, this was not observed. Only 22 DEPs were identified between subjects on prednisone and those not receiving treatment at study entry, compared to over 400 DEPs observed between MG subjects and healthy controls, regardless of prednisone use (Supplemental Fig. 1). These findings align with those of Nelke and colleagues, who reported that, in a highly heterogeneous treated cohort of MG patients, proteomic differences were greater between patients and a small control group (n = 10) than among the four patient consensus clusters (32). Despite the widespread and long-standing use of corticosteroids across a range of diseases, surprisingly little is known about their effects on gene and protein expression following chronic administration. Serum proteomic changes are presumed to result from the genomic actions of corticosteroids, which are themselves modulated by physiological adaptations, including downregulation of the glucocorticoid receptor and feedback regulation of downstream pathways (33). Moreover, glucocorticoid effects on tissue may not be reflected in the circulating proteome, as our findings suggest.

The proteomic signatures differences were a function of MG. Pathway analyses revealed alterations in cytoskeletal and organelle organization. The explanation for such changes in circulation may lie in injury to the large number of neuromuscular junctions across the body. AChR antibodies induce complementmediated injury and deposits of immunoglobulins, complement components, membrane-attack complex, and AChR are found in the synaptic cleft. Macrophage infiltration is not appreciated and therefore, these components and others could make their way into circulation. Complement components of the membrane attack complex are appreciated to be shed with tissue trauma, as would occur in MG, and these could have a pro- or anti-inflammatory effect (34). The same can be said for mechanisms of modulating effects on lymphocytes. Immune cells must migrate from blood to various lymphoid organs and other tissues. KEGG pathway analysis identified leukocyte transendothelial migration, B cell receptor signaling pathway, and Fc receptor pathways to be reduced in the MG subjects (Fig. 2B). Similarly, GSEA evaluation of the GO-BP database was supportive of these results, but also found immunoglobulin production and complement activation were significantly downregulated in MG patients compared to controls. Complement-mediation of disease pathology of MG is well appreciated (35, 36). T cell receptor signaling pathway was significantly upregulated (Fig. 2D). These results may seem counterintuitive that these pathways would be reduced in an active autoimmune disorder; however, this would represent a mechanism to suppress global immune activity in the context of the autoimmune reaction. T cell receptor signaling pathway was significantly upregulated, which could be a suppressor activity. Similar pathways were identified in our transcriptional profiling analysis of thymus obtained from the MGTX study (37).

Consistent with pathway analysis, the most significantly expressed DEPs were related to cytoskeletal function or directly to immune function with several overlapping. TAGLN2, PDLIM2, CNN2, and SEPTIN6 were all originally characterized by cytoskeletal association; their involvement in T cell activation and inflammatory signaling (38–40) make them particularly interesting proteins. CSK, or c-Src terminal kinase, is involved in T cell and B cell receptor signaling and can be found in exosomes (41, 42). Many of the proteins identified are highly likely to be carried in exosomes, which are known to be secreted by a number of cell types in response to stress signals to promote tissue healing (43).

### Machine Learning Assessment

To robustly capture relationships between serum proteins and clinical improvement, we applied a panel of machine learning algorithms, each with distinct pattern sensitivities. First, we used Boruta analysis to identify features that *collectively* contributed to the prediction of treatment response. In our study, features are proteins. Proteins that do not improve the model’s predictive performance are not selected. Importantly, proteins that may be part of the same biological pathway might not all be selected if their inclusion would not provide additional predictive value beyond what is already captured by other selected proteins. Proteins identified through feature selection were utilized in several machine learning algorithms to assess the AUC of the proteins predicting outcomes. This assumption-agnostic approach, combined with nested cross-validation, enabled head-to-head model comparison and revealed Random Forests as optimal for PA-treated patients and XGBoost for those receiving ETTX; each performed excellently with an AUC > 0.9.

Random Forests and XGBoost often outperform probabilistic classifiers (Naïve Bayes, Logistic Regression) and regularized linear models (LASSO, Elastic Net) in complex biological datasets, including large proteomic data sets. This finding lies in the nonlinearity of proteomic data which is further complicated by the correlation of many proteins. XGBoost, is also tree-based ensembles that use boosting. Unlike Random Forests, where trees are built independently, boosting builds trees sequentially, with each new tree focusing on correcting the mistakes made by the previous ones. This allows the model to gradually improve its performance by learning from its own errors. XGBoost builds on this idea by adding regularization to penalize overly complex models, pruning to eliminate unhelpful splits, and parallelization to speed up training.

The eight models represent a spectrum of complexity, underlying model assumptions, and learning strategies, which allowed us to systematically evaluate predictive performance across our proteomic datasets. The use of multiple ML algorithms is critical in biomarker discovery, where signals are often heterogeneous and no single method can guarantee optimal performance (44, 45). The multi-model approach not only increases confidence that our findings were not artifacts of any one modeling assumption and provides insight into the nature of the predictive structure. Overall, these differences highlight how proteomic landscapes shape clinical response in a treatment-dependent manner, with additive effects characterizing PA subjects and nonlinear interactions driving improvement in those receiving ETTX.

### Previous proteomic evaluations in myasthenia gravis

Our study is among the first to utilize both proteomics and ML modeling to predict short-term treatment response in patients with MG. The number of studies utilizing machine learning on biospecimen datasets, not just clinical data, from patients with MG without thymoma is sparse. This includes studies identifying biomarkers from the gut microbiome of MG using metabolomics (12) and rRNA sequencing (13), from an MG monozygotic twin microarray dataset (8, 10), and from proteomic data from AChR-ab positive MG patients (11).

A recent study by Lin et al also utilized LC-MS DIA proteomics to assess pre-surgical differences in serum between effective and ineffective thymectomy in MG; however, samples were from a cohort of patients with thymoma MG (46). Even so, they reported that the protein SELL was downregulated in treatment-responsive thymectomized patients at baseline. This finding is partially in line with our study, in which SELL was considered an important protein in predicting treatment response specifically in patients receiving a thymectomy and prednisone (ETTX patients), albeit the relative expression in ETTX patients who improved was higher than those that did not (Table 1). SELL (L-selectin) has been strongly implicated for its role in the adhesion and trans endothelial migration of leukocytes, especially neutrophils, before being cleared via ectodomain shedding, acting as a marker for neutrophil activation (47, 48).

Five studies used targeted (i.e., O-link) proteomics to investigate changes between MG and HC samples (49–53). In agreement with results reported by Bhandage et al, 2024 and Molin et al, 2017, we found elevated levels of the pro-inflammatory calcium binding protein SA100-A12 in MG patients compared to controls, which may be indicative of increased neutrophil activation and lymphocyte recruitment (54, 55).

#### Proteomic Signature of Prednisone Treatment Effect.

DEPs at baseline were identified that were associated with improved QMG after 6 months and these were distinct for ETTX and PA groups, despite the well-matched clinical characteristics of these subjects. Seven proteins (Table 1) were associated with treatment response, with CCL16 and IGHV3–43 being the most clearly related to the immune response. CCL16 is a chemokine primarily expressed in the liver but also the thymus and some lymphocytes. CCL16 is appreciated as being responsible for recruitment of T cells to sites of pathology and other chemokines are well-established to be involved in MG, this has not been appreciated before for CCL16 (56). IGHV3–43 is a portion of the variable region of the heavy chain of IgG, which would be expected to be detected in serum, but why the lower level of this specific IgG heavy chain in serum would be associated with improvement is not clear. However, given that genetic variations in the immunoglobulin heavy chain locus may drive antibody response could suggest this IGHV3–43 may be of particular importance in MG (57). Results from the GeneAgent analysis similarly identified the immune response roles, and a mild predominance of ribosomal involvement by RPS3 and RPS3A.

#### Proteomic Signature for Thymectomy plus Prednisone Treatment Effect.

The 17 proteins identified by Boruta analysis as predictive of clinical improvement in MG patients treated with thymectomy plus prednisone likely reflect a coordinated biological response to immune modulation and tissue remodeling. These proteins encompass key processes including immune regulation and complement control (CD55, SELL, IGKV1–5, RNASET2), extracellular matrix and stromal reorganization (MMP3, FBLN5, CCN5), cytoskeletal dynamics and cellular trafficking (ARHGAP9, RAB14, RDX), stress response and protein repair (HSP90AA1, PCMT1), and modulation of the BMP/TGF-β axis involved in thymic epithelial architecture (CHRDL1, SPP2). Collectively, these proteins may serve as systemic indicators of thymic regression, loss of germinal center activity, suppression of autoreactive B and T cell signaling, and the resolution of inflammatory and structural abnormalities that drive disease activity. Their predictive value likely lies in their ability to capture early molecular changes that precede and enable long-term clinical benefit as observed in the MGTX trial. From a translational standpoint, the biological roles of these proteins suggest potential therapeutic targets or mechanistic entry points for understanding how thymectomy induces systemic immune reprogramming.

SVEP1 had the greatest SHAP value predictive of improvement (Fig. 6D, E). SVEP1 (Sushi, von Willebrand factor type A) is a large extracellular matrix protein that has been identified to be associated with poor outcomes in several conditions including coronary artery disease, dementia, and hypertension and with poor outcomes in heart failure, where it is strongly associated with activated T cells (58–60). SVEP1 is composed primarily of complement control protein (CCP, also known as Sushi) domains; whether it binds complement proteins is not known. Elevated SVEP1 levels in blood are also associated with diseases of aging, and SVEP1 has been shown to activate the mTOR pathway, a key regulator of longevity. Given autoimmune disorders increase with chronological age, these findings provide the intriguing insight that these early-onset MG patients have an advanced biological age.

Heat shock protein 90α (Hsp90α) is a ubiquitously expressed heat shock protein, which interacts with close to two thousand proteins and numerous biological pathways (61, 62). Elevations in serum are associated with greater cancer risk and vascular inflammation. Hsp90α has been shown to suppress or activate immune processes depending on specific conditions. SPP2 is produced in the liver and known to be found in circulation. It binds and activates intracellular signaling pathways of the TGF-beta superfamily cytokines. Interestingly, SPP2 shares sequence homology with osteopontin. Osteopontin gene polymorphisms are associated with treatment response to prednisone in MG (63). CD55 is a complement inhibitory protein known to be concentrated at the neuromuscular junction and protective of injury to antibody-mediated, complement-induced in experimental MG (64). While typically found on cell surfaces, CD55 exists in soluble form and in culture reduces proliferation peripheral blood monocytes and would be expected to modulate complement activity (65, 66). CD55 has four CCP domains and therefore shares structural similarities to SVEP1.

The thymus of patients with MG caused by AChR antibodies demonstrates thymic hyperplasia characterized by lymphoid follicles and germinal center formation, which was documented for the present group of patients (67). The germinal centers are a source of AChR-specific B cells and contain autoreactive T cells. Removal of the thymus reduces a source of autoreactive T and B cells but, despite its removal, clinical disease persists in a variable number of patients.

The panel of proteins predictive of outcome in ETTX patients supports a fundamental difference in individual susceptibility to removal of thymus. The proteins identified and our approach to this question reveals that a single factor, such as T regulatory cell dysfunction, as appreciated in patients with MG, is unlikely to be the single answer to treatment effect of thymectomy. GeneAgent results supported critical roles of cell trafficking in these proteins; this would be consistent with the susceptibility of such pathways to treatment outcome based on the exodus of pathogenic cells from the thymus to peripheral immune organs.

### Clinical Impact

The clinical importance of our findings lies in the identification of serum protein signatures associated with treatment response to thymectomy plus prednisone and prednisone alone, offering a potential tool for personalized treatment planning in myasthenia gravis. Currently, decisions regarding thymectomy are based on generalized criteria without reliable biomarkers to predict individual benefit (2). Our results suggest that proteomic profiling could enable clinicians to identify patients more likely to respond favorably to thymectomy, thereby reducing unnecessary surgeries in non-responders, expediting treatment decisions for those likely to benefit, and guiding others toward alternative therapies. The observed heterogeneity in treatment response is consistent with findings by Nelke et al., who identified patient subgroups with differential responsiveness, including one enriched for complement inhibition benefit (32). Further, the prednisone-only related signature would identify poor responders to the standard of care treatment for MG (68). This approach parallels precision oncology strategies, where surgical or pharmacologic interventions are tailored using molecular markers. Moreover, stratification based on this proteomic signature could inform the development of novel therapeutic targets for nonresponders.

## Conclusions

Baseline serum proteomics distinguished treatment-specific predictors of clinical response in patients with MG. PA-patients’ response was explained mainly by single protein markers, whereas ETTX-patients’ response was predicted by complex protein interactions. These findings represent an important first step toward biomarker-guided treatment in MG; with validation in independent cohorts, proteomic signatures could refine patient stratification, inform clinical trial design, and enable more personalized therapeutic decisions.

## Supplementary Material

Supplementary Files

This is a list of supplementary files associated with this preprint. Click to download.

- AdditionalFile1.pdf
- AdditionalFile2.pdf
- AdditionalFile3.pdf
- AdditionalFile4.pdf
- AdditionalFile5.xlsx
- AdditionalFile6.pdf
- AdditionalFile7.pdf

## Figures and Tables

**Table 1:** Baseline proteins predictive of clinical improvement in PA and ETTX treated MG patients.

MG Group	Protein	Improved	Not Improved	p-value
PA	CCL16	7.05 ± 0.32	8.13 ± 0.39	0.04
	CILP2	12.08 ± 0.12	11.61 ± 0.15	0.017
	GALNT1	9.30 ± 0.30	10.27 ± 0.12	0.0046
	IGHV3–43	10.73 ± 0.22	11.30 ± 0.16	0.04
	RPS3	8.42 ± 0.35	7.05 ± 0.47	0.025
	RPS3A	8.61 ± 0.42	7.01 ± 0.17	0.0012
	VAPB	11.46 ± 0.23	11.95 ± 0.10	0.057
ETTX	ARHGAP9	7.34 ± 0.34	8.35 ± 0.46	0.086
	CCN5	9.47 ± 0.42	8.33 ± 0.78	0.21
	CD55	6.34 ± 0.22	7.73 ± 0.49	0.019
	CHAD	13.06 ± 0.15	12.38 ± 0.21	0.015
	CHRDL1	9.19 ± 0.31	7.96 ± 0.51	0.051
	FBLN5	10.79 ± 0.22	9.86 ± 0.36	0.038
	HS1BP3	7.61 ± 0.27	8.83 ± 0.44	0.027
	HSP90AA1	9.19 ± 0.42	10.70 ± 0.32	0.0068
	IGKV1–5	19.85 ± 0.11	19.61 ± 0.19	0.30
	MMP3	12.87 ± 0.21	13.76 ± 0.31	0.026
	PCMT1	8.49 ± 0.34	7.88 ± 0.67	0.42
	RAB14	10.18 ± 0.36	8.96 ± 0.63	0.11
	RDX	10.34 ± 0.24	10.70 ± 0.39	0.45
	RNASET2	11.03 ± 0.07	10.81 ± 0.16	0.21
	SELL	14.01 ± 0.09	13.72 ± 0.17	0.14
	SPP2	15.20 ± 0.13	15.76 ± 0.14	0.0059
	SVEP1	11.06 ± 0.12	10.02 ± 0.26	0.002

Values reported as log_2_ abundances (mean ± SEM). P-values are reported from students two-tailed ttests between improved and not improved groups.

**Table 2:** Performance metrics across 5 outer folds of PA patients.

Metric	NB	Logistic	LASSO	EN	CT	RF	Ada	XGB
AUC	*0.87 ± 0.07*	0.82 ± 0.07	0.87 ± 0.03	0.87 ± 0.03	0.64 ± 0.09	**0.91 ± 0.06**	0.74 ± 0.08	0.82 ± 0.09
AUCPR	0.95 ± 0.03	0.92 ± 0.03	0.94 ± 0.02	0.94 ± 0.02	0.75 ± 0.07	0.95 ± 0.03	0.92 ± 0.03	0.90 ± 0.06
Prec	0.90 ± 0.07	0.79 ± 0.09	0.80 ± 0.09	0.79 ± 0.07	0.71 ± 0.10	0.86 ± 0.07	0.83 ± 0.07	0.77 ± 0.08
Recall	0.68 ± 0.10	0.68 ± 0.09	0.80 ± 0.06	0.89 ± 0.05	0.72 ± 0.06	0.92 ± 0.05	0.77 ± 0.08	0.84 ± 0.09
Acc	0.76 ± 0.05	0.68 ± 0.03	0.73 ± 0.05	0.78 ± 0.05	0.64 ± 0.09	0.85 ± 0.06	0.76 ± 0.08	0.76 ± 0.07
Spec	0.88 ± 0.07	0.70 ± 0.18	0.65 ± 0.19	0.62 ± 0.12	0.47 ± 0.18	0.73 ± 0.11	0.72 ± 0.13	0.62 ± 0.12
F_1_	0.76 ± 0.07	0.71 ± 0.06	0.78 ± 0.04	0.83 ± 0.04	0.71 ± 0.07	0.88 ± 0.06	0.80 ± 0.07	0.80 ± 0.08
MCC	*0.56 ± 0.09*	0.42 ± 0.10	0.52 ± 0.10	0.52 ± 0.10	0.17 ± 0.20	**0.68 ± 0.12**	0.47 ± 0.17	0.47 ± 0.14

Metrics reported as mean ± SEM. The best performing AUC and MCC metrics are in bold and the runner up in italics. NB, Naïve Bayes; EN, elastic net; CT, classification tree; RF, random forest; Ada, adaptive boosting; XGB, extreme gradient boosting; AUC, area under the ROC curve; AUCPR, area under the precision-recall curve; Prec, precision; Acc, accuracy; Spec, specificity; F_1_, F-score (harmonic mean of precision and recall); MCC, Matthew’s correlation coefficient.

**Table 3:** Performance metrics across 5 outer folds of ETTX patients.

Metric	NB	Logistic	LASSO	EN	CT	RF	Ada	XGB
AUC	0.83 ± 0.05	0.66 ± 0.13	0.90 ± 0.08	0.90 ± 0.08	0.56 ± 0.08	0.90 ± 0.06	*0.95 ± 0.05*	**0.99 ± 0.01**
AUCPR	0.92 ± 0.05	0.89 ± 0.05	0.96 ± 0.03	0.96 ± 0.03	0.78 ± 0.05	0.96 ± 0.02	1.00 ± 0.00	1.00 ± 0.00
Prec	0.80 ± 0.07	0.84 ± 0.09	0.90 ± 0.05	0.90 ± 0.05	0.76 ± 0.07	0.79 ± 0.10	0.95 ± 0.03	0.88 ± 0.06
Recall	0.88 ± 0.07	0.76 ± 0.08	0.92 ± 0.05	0.92 ± 0.05	0.70 ± 0.11	0.97 ± 0.02	1.00 ± 0.00	1.00 ± 0.00
Acc	0.76 ± 0.06	0.71 ± 0.08	0.89 ± 0.04	0.89 ± 0.04	0.58 ± 0.05	0.78 ± 0.08	0.96 ± 0.03	0.91 ± 0.04
Spec	0.41 ± 0.19	0.58 ± 0.19	0.79 ± 0.10	0.79 ± 0.10	0.42 ± 0.19	0.44 ± 0.23	0.83 ± 0.11	0.75 ± 0.10
F1	0.83 ± 0.05	0.78 ± 0.06	0.91 ± 0.04	0.91 ± 0.04	0.69 ± 0.04	0.85 ± 0.06	0.97 ± 0.02	0.93 ± 0.04
MCC	0.40 ± 0.24	0.37 ± 0.19	0.73 ± 0.08	0.73 ± 0.08	0.08 ± 0.17	0.66 ± 0.20	**0.88 ± 0.07**	**0.81 ± 0.08**

Metrics reported as mean ± SEM. The best AUC and MCC metrics are in bold and the runner ups are in italics.

## Data Availability

The R code used to run nCV is available on GitHub at https://github.com/drkgil/NestCV. The datasets used and analyzed during the current study are available from the corresponding author on reasonable request.

## Abbreviations

MG: Myasthenia gravis
MGTX: Thymectomy Trial in Non-Thymomatous Myasthenia Gravis Patients Receiving Prednisone Therapy
AChR: acetylcholine receptor
ETTX: thymectomy plus prednisone
PA: prednisone alone
QMG: Quantitative MG
LC-MS: liquid chromatography-mass spectrometry
ML: machine learning
IRB: institutional review board
DDA: data dependent acquisition
DIA: data independent acquisition
DEP: differentially expressed protein
HC: healthy control
ORA: over representation analysis
GSEA: gene set enrichment analysis
nCV: nested cross validation
LASSO: least absolute shrinkage and selection operator
CART: classification and regression tree algorithms
AdaBoost, Ada: adaptive boosting
XGBoost: extreme gradient boosting
PCA: principal component analysis
GO-BP: gene ontology biological processes
KEGG: Kyoto Encyclopedia of Genes and Genomes
SEM: standard error mean
NB: Naïve Bayes
EN: elastic net
CT: classification tree
RF: random forest
AUC: area under the ROC curve
ROC: receiver operating characteristic
AUCPR: area under the precision-recall curve
MCC: Matthew’s correlation coefficient
SHAP: Shapley additive explanations
CCL16: C-C motif chemokine ligand 16
CILP2: cartilage intermediate layer protein 2
GALNT1: polypeptide N-acetylgalactosaminyltransferase 1
IGHV3–43: immunoglobulin heavy variable 3–43
RPS3: ribosomal protein S3
RPS3A: ribosomal protein S3A
VAPB: VAMP (vesicle-associated membrane protein)-associated protein B and C
ARHGAP9: Rho GTPase activating protein 9
CCN5: cellular communication network factor 5
CD55: decay accelerating factor for complement
CHAD: chondroadherin
CHRDL1: chordin like 1
FBLN5: fibulin 5
HS1BP3: HCLS1 binding protein 3
HSP90AA1: heat shock protein 90 alpha family class A member 1
IGKV1–5: immunoglobulin kappa variable 1–5
MMP3: matrix metallopeptidase 3
PCMT1: protein-L-isoaspartate (D-aspartate) O-methyltransferase
RAB14: member RAS oncogene family, F protein-binding protein 1
RDX: radixin
RNASET2: ribonuclease T2
SELL: selectin L, lymphocyte adhesion molecule 1
SPP2: secreted phosphoprotein 2
SVEP1: sushi, von Willebrand factor A, EGF and pentraxin domain containing 1
